# Anxiety and depression symptoms among women attending group-based patient education courses for hereditary breast and ovarian cancer

**DOI:** 10.1186/s13053-016-0062-5

**Published:** 2017-01-11

**Authors:** Wenche Listøl, Hildegunn Høberg-Vetti, Geir Egil Eide, Cathrine Bjorvatn

**Affiliations:** 1 Western Norway Familial Cancer Center, Haukeland University Hospital, Bergen, Norway; 2 Center for Medical Genetics and Molecular Medicine, Haukeland University Hospital, Bergen, Norway; 3 Centre of Clinical Research, Haukeland University Hospital, Bergen, Norway; 4 Learning and Mastery Center in Bergen, Bergen, Norway; 5 Department of Clinical Science, University of Bergen, Bergen, Norway; 6 Department of Global Public Health and Primary Care, University of Bergen, Bergen, Norway

**Keywords:** Group-based patient education course, Genetic counseling, Hereditary cancer, Anxiety symptoms, Depression symptoms

## Abstract

**Background:**

Women carrying *BRCA*-mutations are facing significant challenges, including decision making regarding surveillance and risk-reducing surgery. They often report that they are left alone with these important decisions. In order to enhance the genetic counselling session we organized a group-based patient education (GPE) course for women with *BRCA*-mutations. The study aims were to characterize women attending a group-based patient education (GPE) course for hereditary breast and ovarian cancer, consider the usefulness of the course, evaluate symptoms of anxiety and depression among the participants, and finally investigate whether their levels of anxiety and depression changed from before to after the course session.

**Methods:**

A prospective study was conducted. Two weeks before (T1) and 2 weeks after (T2) attending the GPE-course the participants received questionnaires by mail. We collected information on demographic- and medical variables, anxiety and depression using Hospital Anxiety and Depression Scale (HADS), self-efficacy using The Bergen Genetic Counseling Self-Efficacy scale (BGCSES) and coping style using the Threatening Medical Situations Inventory (TMSI). A total of *N* = 100 (77% response rate) women participated at baseline and 75 (58% response rate) also completed post-course assessment.

**Results:**

The mean level of anxiety symptoms was elevated among participants but decreased significantly during follow-up. Lower anxiety symptom levels were associated with “longer time since disclosure of gene test result”, “higher levels of self-efficacy” and having experienced “loss of a close relative due to breast or ovarian cancer”. Lower depression symptom levels were associated with “higher levels of education” and “loss of a close relative due to breast or ovarian cancer”.

**Conclusion:**

The women in this study seemed to benefit from the GPE course. Women newly diagnosed with a *BRCA* mutation who reported lower levels of self-efficacy and lower levels of education were more vulnerable. These women need special attention.

## Background

Carriers of mutations in the *BRCA1-* or *BRCA2* gene (breast cancer genes) have a substantially increased risk of both breast and ovarian cancer. The cumulative risk for breast cancer by age 70 has been reported to 45–60% for *BRCA1* mutation carriers and 27–55% for *BRCA2* mutation carriers with a corresponding risk for ovarian cancer of 31–59% and 6–16.5% for *BRCA1* and *BRCA2* mutation carriers, respectively [1, 2]. Beginning at age 25, women with a mutation in either of these genes are offered breast cancer surveillance, including annual mammography and breast magnetic resonance imaging (MRI). Between 35 and 40 years, risk-reducing salpingo-oophorectomy is recommended [3, 4]. A growing number of women are also opting for risk-reducing bilateral mastectomy [5–8]. Decisions to have risk-reducing surgeries are both irreversible and existential in nature; they are also associated with both psychosocial burden as well as hope for a longer and healthier life [3, 9, 10].

Genetic counseling is a specialized healthcare service provided by specialists in medical genetics and genetic counselors. It is described as a communication and educational process that deals with the challenges associated with the occurrence, or the risk of occurrence, of a genetic disorder within a family [11]. In the counseling session, it is important to impart information that provides the basis for informed decisions regarding gene testing, surveillance programs, and prophylactic surgery. It is also critical to acknowledge that the genetic information may have a significant impact on the patient’s extended family. The counseling session may trigger ethical dilemmas such as whom should be informed and when the best time may be to pass on the genetic information to children and other family members [6, 12]. Thus, patients need proper counseling and follow-up to make decisions related to these aspects [13]. Genetic counseling is often a one-on-one consultation. Previous research has shown that group-based patient education (GPE) courses are valuable toward empowered decision-making among women [14–17] and may serve as a useful supplement to traditional individual counseling.

Over the past decades, several studies have focused on emotional distress among patients seeking genetic counseling for hereditary cancer [6, 12, 18]. Anxiety is a normal reaction to a stressor. It is most often described as the emotion of fear involving feelings of tension, nervousness, apprehension, worry and dread for something perceived as threatening in the future [19]. Mild to moderate anxiety symptoms are vague and unsettling, while severe anxiety symptoms can be extreme and have a serious impact on daily life. Depression has been defined as an emotion of sadness, with feelings of sorrow, hopelessness, gloom, lack of energy and initiative [20]. Anxiety and depression are different conditions, but they commonly occur together [21].

Many factors influence a *BRCA* mutation carrier’s anxiety and depression levels. Earlier studies have shown that younger age, having children, and experience of breast and ovarian cancer among close relatives are strong predictors of high emotional distress [22, 23]. Self-efficacy and social support seem to play important roles in reducing anxiety and depression levels among patients at risk for hereditary cancer [18]. Previous studies revealed that patients are often satisfied with genetic counseling sessions [24, 25]. Providing medical information and emotional support appears to be important for increasing patients’ satisfaction [12, 26]. Several studies have also shown that a monitoring coping style is related to reduced psychological distress in genetic counseling for hereditary cancer [27, 28]. Other studies have shown that some individuals need additional counseling or different interventions such as GPE courses [14–17, 25, 29].

Patient education is a major task for healthcare workers and is incorporated in formal legislation or regulations in several countries [30, 31]. Educating patients and their significant others is considered a part of patient treatment and has been proven to increase compliance with treatment [32]. Learning and Mastery Centers have been established to support the health services with education courses for both patients and health professionals [33]. Previous research indicates a positive effect of GPE courses on psychosocial outcomes such as mental health, coping, and knowledge about their illness [32, 34–36]. Group intervention appears to be relevant and highly acceptable to women with a mutation in one of the *BRCA* genes [8, 16, 17]. Participating in a patient education group seems to help these women make cancer risk management decisions [37]. GPE for *BRCA* mutation carriers may be the ideal forum for exploring challenges, such as when to share genetic information with the extended family and dealing with the guilt of having passed the mutation on to their children [16]. Interacting with other women with a *BRCA* mutation also gives patients the opportunity to learn from each other and reinforces a feeling of not being alone [14].

The main goal of the study was to describe the *BRCA* mutation carriers who attended a GPE course. We also investigated whether the GPE course was experienced as a useful intervention for these women. Finally, we evaluated the characteristics of those with increased levels of anxiety or depression symptoms and whether these symptoms changed from before the course until after.

## Methods

### Study design and procedures

A prospective study was undertaken in women with *BRCA* mutations who took part in a GPE course. Two weeks before attending the course, the women received written information about the study, were invited to provide written informed consent and were provided with the first questionnaires (T1). Two weeks after the course (T2), follow-up questionnaires were mailed to those who had provided written informed consent. The Data Protection Official for Haukeland University Hospital approved the study.

### Study sample

From October 2011 to August 2013, eight GPE courses were arranged. Both healthy women and women with a personal history of cancer were invited to participate in a course if they fulfilled the following inclusion criteria: over 18 years old, able to read Norwegian, had been to genetic counseling for hereditary breast and ovarian cancer, and were carrying a *BRCA* mutation. Of the 160 women invited, 130 signed up for a GPE course and received a confirmation phone call from the staff at the Center for Medical Genetics, Haukeland University Hospital.

### The group-based patient education (GPE) course

To meet the women’s needs for psychosocial support after genetic counseling and a positive *BRCA* test result, our GPE course was based on earlier proposals from groups associated with the Norwegian Cancer Society. The GPE course was standardized to a seven-hour session, with a maximum of 20 participants in each course. The primary aims of the course were to support and empower participants to live their lives and make decisions based on information and advice from health professionals, and to provide the opportunity for them to learn from each other. We incorporated a strong user perspective in the planning, implementation, and evaluation of the GPE course. This ensured that the users’ voice was heard and increased the chances of keeping the course patient-focused [33]. See Table 1 for an overview of the themes included in the GPE course.

**Table 1:** Thematic overview of the GPE course

• User perspective of a women carrying a *BRCA* mutation who is an “expert by experience” ^a^	
• General information about hereditary breast and ovarian cancers and the consequences of being a mutation carrier, including the family perspective	
• Information about risk-reducing bilateral salpingo-oophorectomy and hormone replacement therapy	
• Medical information about prophylactic bilateral mastectomy	
• Discussions about body image and sexuality	
• Information from patient networking groups	
• Interaction between the course participants in small groups, which gives them an opportunity to learn from each other and reinforce a feeling of not being alone	

^a^ “Expert by experience”: a *BRCA* carrier who received her gene test results several years ago

### Study measurements

#### Sociodemographic and medical variables

The sociodemographic and medical variables included: age, marital status, children, education, cancer or another chronic disease, and time since the genetic test was carried out. We also collected family history of breast and ovarian cancer and asked whether the respondents had experienced loss of significant others.

### Standardized evaluation following the GPE course

We used a standardized evaluation developed by the Norwegian National Advisory Unit on Learning and Mastery in Health. It is designed to support quality improvement (NK LMH 2009).

### Situation specific self-efficacy

The Bergen Genetic Counseling Self-efficacy Scale (BGCSES) was developed according to Bandura’s guidelines for constructing self-efficacy scales (revised 2001) (Albert Bandura, Stanford University Palo Alto, CA, USA). According to Bandura self-efficacy is a person’s beliefs in own ability to cope with different challenges and to execute some control over environmental events. It was developed by a panel of medical geneticists, genetic counselors and psychologists [18]. The scale consists of 20 items describing tasks and challenges likely to occur during a GPE course, and the individual’s belief in their ability to cope with different challenges. Each item is rated on a scale from 1 (cannot do at all) to 11 (can do without difficulty). The average score of BGCSES for each individual (range 1–11) was used in the present study. Higher scores indicate higher self-efficacy. The reliability of the scale, estimated by Cronbach’s alpha, was 0.85.

### Symptoms of anxiety and depression

The Hospital Anxiety and Depression Scale (HADS) was used to assess symptoms of anxiety and depression. The questionnaire has two subscales each with seven items that measure symptoms of anxiety and depression, respectively. Each item is scored on a four-point scale. Each total subscale score ranges from 0 to 21 [38]. A score of eight or higher was used as the cut-off for elevated symptoms of both anxiety and depression [39]. In the present study, reliability values for the HADS anxiety and depression subscales, estimated with Cronbach’s alpha, were 0.87 and 0.84, respectively.

### Coping style

The Threatening Medical Situation Inventory (TMSI) was used to measure two cognitive coping styles in the domain of medical threat: monitoring (confrontation style) and blunting (avoidance style). The TMSI includes four descriptions of threatening medical situations. Each TMSI situation is followed by three monitoring and three blunting items, arranged in random order, which are scored on a five-points scale from 1 (not at all applicable to me) to 5 (strongly applicable to me). Total monitoring and blunting scores were calculated by summing the relevant items. Ranges for each subscale are 12–60 [40]. Consistent with several other studies, in the present study we used only the monitoring subscale [27, 41]. Reliability of the TMSI monitoring subscale, estimated by Cronbach’s alpha, was 0.79.

### Statistical analyses

Descriptive statistics were used to describe the distributions of the socio-demographic and medical variables (i.e. the mean, standard deviation (SD) and proportions). Paired-sample *t*-tests were used to compare means of repeated measurements and changes in values for anxiety and depression symptom subscores were analyzed with McNemar’s exact test. To test for differences between the final study sample and those who dropped out from T1 to T2, we used an independent *t*-test for continuous variables, and for categorical variables we calculated the Fisher’s exact mid *p*-value [42].

To identify the characteristics related to HADS anxiety and HADS depression, the subscale scores were regressed on the selected predictor variables using a mixed linear model module. The mixed linear model uses all available data and can account for correlations between repeated measurements on the same subjects and has sufficient flexibility to model time effects [43]. All predictors were entered into the mixed linear models to assess both their main effects and their possible interactions with time. The regression analyses were run backwards stepwise, both with and without interaction with time.

Missing values were replaced according to the guidelines for each instrument or by the individual’s own average score for each questionnaire when more than 50% of the items were completed. Reliability was estimated by Cronbach’s alpha for all scales used in this study. A two-tailed significance level of 0.05 was used. All data were analyzed using SPSS version 22.

## Results

### The study sample and characteristics of predictor variables

Of the 130 eligible women, 100 (77%) consented to the study and returned their questionnaires at baseline, and 75 (58%) completed both questionnaires. We have no data on the 30 non-responses. Characteristics of sociodemographic and medical variables for the study sample are provided in Table 2. The mean age of the study sample was 45.8 years (range: 26–69 years) and the mean time since they received the *BRCA* test results was 5.3 years (range: 1 month–15 years). About 87% were cohabiting, 87% had children, and 89% reported their education level to be high school or above. Among the participants, 26% had a personal history of cancer. The majority of the samples had experienced cancer and death among their close relatives. The participants reported both high situation specific self-efficacy and high average score for monitoring coping style (Table 3).

**Table 2:** Sociodemographic and medical characteristics of women attending group-based patient education (GPE) course

Characteristic		Respondents (*n* = 100)		Drop-outs^a^ (*n* = 25)		
	Category	*n*	%	*n*	%	*p*-value
Marital status						0.083^b^
	Married/cohabiting	87	87.0	19	21.8	
	Living alone	13	13.0	6	3.3	
Children						0.026^b^
	Yes	87	87.0	20	21.8	
	No	13	13.0	5	3.3	
Educational status						0.501^c^
	Primary school	11	11.0	1	2.8	
	High school	39	39.0	11	9.8	
	University	50	50.0	13	12.5	
Breast cancer or ovarian cancer						0.451^b^
	Yes	26	26.0	5	6.5	
	No	74	74.0	20	18.5	
FDR and/or SDR died due to BOC						0.643^b^
	Yes	83	83.0	20	20.8	
	No	17	17.0	5	4.3	
Loss of significant others						0.785^b^
	Yes	64	64.0	15	16.0	
	No	36	36.0	10	9.0	
		mean (SD)		mean (SD)		*p*-value^d^
Age, *years*		47.7 (12.6)		39.9 (8.2)		0.005
Years since received BRCA1 or BRCA2 mutation result		5.0 (4.4)		6.3 (3.9)		0.182
HADS anxiety (range: 0–21)		6.1 (4.3)		6.3 (3.8)		0.814

*Abbreviations*: *FDR* first degree relative, *SDR* second degree relative, *BOC* Breast or ovarian cancer, *SD* standard deviation, *BRCA1* breast cancer type 1 susceptibility protein, *BRCA2* breast cancer type 2 susceptibility protein, *HADS* Hospital Anxiety and Depression Scale

^a^ Drop-outs are defined as having answered on baseline questionnaires and not on the follow-up

^b^ Fisher’s exact 2-sided mid-*p* value (calculated from output of chi-square test)

^c^ Chi-square 2-sided linear-by-linear association test

^d^ Independent samples *t*-test

**Table 3:** Study measurement and descriptive statistics in women attending group-based patient education (GPE) course

Measure	T1 = 2 weeks before GPE course				T2 = 2 weeks after GPE course				
	*N*	Mean	SD	%	*N*	Mean	SD	%	*p*-value
HADS-anxiety (range: 0–21)	100	6.2	4.1		74	5.2	3.9		0.003 ^a^
HADS-A score ≥ 8	29			29.0	14			18.9	0.065 ^b^
HADS-depression (range: 0–21)	100	2.8	3.3		74	2.6	3.2		0.211 ^a^
HADS-D ≥ 8	10			10.0	6			8.1	1.000 ^b^
TMSI-monitors (range: 12–60)	99	42.8	7.4						
Situation specific self-efficacy (range: 1–11)	100	8.9	1.4						

*Abbreviations*: *SD* standard deviation, *HADS* Hospital Anxiety and Depression Scale, *TMSI* Threatening Medical Situations Inventory

^a^ Paired samples *t*-test

^b^ McNemar’s exact test

On average, the respondents reported that they were satisfied with the GPE course. Each theme in the GPE course was scored on a five-point scale from 1 (“not important”) to 5 (“very important”). The average score for all themes was 3.84 (SD: 0.08). Of the 75 women who responded to the follow-up questionnaires, 41.3% reported that the intervention was “good”, and the rest (58.7%) reported it went “very well”. None of the participants indicated that they were dissatisfied with the GPE course. A majority of the women, 75.7%, reported that they had learned something new. However, 34.7% reported that they missed something and 33.3% were unsure if they missed important topics.

### Drop-outs

Drop-outs were defined as having responded at T1 but not at T2. Drop-outs (25%) were significantly younger, with a mean age of 40.0 years compared to 47.7 years among the complete study sample (*p* = 0.005). There were also significantly more drop-outs who reported having “no children” (*p* = 0.026). Otherwise, the drop-outs did not differ on any of the other study variables (Table 2).

### Outcome variable: anxiety (HADS-A) and depression (HADS-D)

Mean score, SDs, and numbers of individuals with symptom scores for anxiety and depression above the cut-off values are provided in Table 3. The mean HADS subscale score for anxiety was 6.2 (SD: 4.14) at T1, with a significant decrease to 5.2 (SD: 3.95) at T2 (*p* = 0.003). The mean HADS-D subscale score was 2.8 (SD: 3.33) and did not change significantly from T1 to T2. The number of participants with scores above the cut-off level of HADS-A dropped from T1 to T2 but the decrease was not statistically significant (*p* = 0.065). The proportion of individuals with a HADS-D score above the cut-off level did not change significantly from T1 to T2 (Table 3).

### Mixed linear model analysis of anxiety and depression symptoms

The following predictors were used: age, having children, cohabiting status, educational status, having cancer, experiencing the death due to breast or ovarian cancer of a first-degree and/or second-degree relative, time since disclosure of the gene test results, monitoring coping style, and situation specific self-efficacy.

The results of the mixed linear model analyzed for HADS-A are provided in Table 4. The final model of the mixed linear model showed that the average level of anxiety symptoms varied with time and that the highest levels were at T1. Anxiety symptom levels decreased with greater time since disclosure of the gene test result, a higher level of situation specific self-efficacy, and if the women had experienced losing a first and/or second-degree relative due to breast or ovarian cancer.

**Table 4:** Mixed linear regression analyses of Hospital Anxiety and Depression Scale, Anxiety in the study sample

Variables	Not adjusted			Fully adjusted (*n* = 97 ^a^)			Final model ^b^ (*n* = 98^a^)		
	*b*	95% CI	*p*-value ^c^	*b*	95% CI	*p*-value^c^	*b*	95% CI	*p*-value ^c^
Intercept (Anxiety)				16.89	(9.45, 24.32)	< 0.001	17.91	(13.04, 22.78)	< 0.001
Educational status (*n* = 100)			0.597			0.331			
University	−1.21	(−3.76, 1.34)		−1.23	(−3.73, 1.27)				
High school	−1.29	(−3.91, 1.32)		−1.50	(−3.99, 0.99)				
Primary school	0.00	(reference)		0.00	(reference)				
Living with someone (*n* = 100)	−0.19	(−2.52, 2.14)	0.872	0.49	(−1.93, 2.90)	0.691			
Children (*n* = 100)	0.30	(−2.02, 2.61)	0.801	1.31	(−1.13, 3.76)	0.289			
BOC (*n* = 100)	1.22	(−0.52, 2.96)	0.167	0.12	(−1.83, 2.06)	0.906			
FDR/SDR died due to BOC (*n* = 100)	−2.62	(−4.61, −0.62)	0.011	−2.06	(−4.12, −0.00)	0.050	−2.25	(−4.08, −0.43)	0.016
Age per 10 years (*n* = 100)	0.03	(−0.61, 0.67)	0.926	0.07	(−0.68, 0.82)	0.862			
Disclosure gene test results/years (*n* = 98)	−0.23	(−0.40, −0.05)	0.012	−0.26	(−0.43, −0.09)	0.003	−0.25	(−0.41, −0.09)	0.002
Monitoring coping style per 10 (*n* = 99)	0.07	(−0.98, 1.12)	0.899	0.35	(−0.63, 1.32)	0.485			
Situation specific self-efficacy (*n* = 100)	−1.02	(−1.55, −0.49)	< 0.001	−1.10	(−1.70, −0.51)	< 0.001	−0.96	(−1.48, −0.45)	< 0.001
Time	−0.91	(−1.47, −0.35)	0.002	−0.90	(−1.46, −0.33)	0.002	−0.92	(−1.48, −0.35)	0.002

*Abbreviations*: *b* estimated regression coefficient, *CI* confidence interval, *FDR* first degree relative, *SDR* second degree relative, *BOC* Breast or ovarian cancer

^a^ Number of subjects having completed the included variables on at least one occasion during the data collection time

^b^ Final model: After backward stepwise selection from fully adjusted model at significance level 0.05

^c^ F-test

The final model of the mixed linear model analyzed for HADS-D is provided in Table 5. In contrast, the average level of depression symptoms did not change from T1 to T2. However, a lower depression score was related to a higher level of education and to having experienced death related to breast and/or ovarian cancer among a first and/or second-degree relative.

**Table 5:** Mixed linear regression analyses of Hospital Anxiety and Depression Scale, Depression in the study sample

Variables	Not adjusted			Fully adjusted (*n* = 97 ^a^)			Final model ^b^ (*n* = 100 ^a^)		
	*b*	95% CI	*p*-value ^c^	*b*	95% CI	*p*-value ^c^	*b*	95% CI	*p*-value ^c^
Intercept (Depression)				9.98	( 3.73, 16.22)	0.002	7.26	(5.03, 9.49)	< 0.001
Educational status (*n* = 100)			0.046			0.033			0.013
University	−2.45	(−4.44, −0.47)		−2.31	(−4.42, −0.19)		−2.54	(−4.43, −0.65)	
High school	−2.36	(−4.40, −0.32)		−2.66	(−4.76, -0.56)		−2.92	(−4.89, −0.96)	
Primary school	0	(reference)		0	(reference)		0	(reference)	
Living with someone (*n* = 100)	−0.24	(−2.10, 1.62)	0.797	0.16	(−1.86, 2.17)	0.877			
Children (*n* = 100)	0.84	(−1.01, 2.68)	0.371	0.87	(−1.19,2.92)	0.404			
BOC (*n* = 100)	1.69	(0.32, 3.05)	0.016	0.73	(−0.90, 2.37)	0.374			
FDR/SDR dead due to BOC (*n* = 100)	−2.25	(−3.83, −0.67)	0.006	−1.85	(−3.58, −0.13)	0.036	−2.59	(−4.17, −1.01)	0.002
Age per 10 years (*n* = 100)	0.43	(−0.07, 0.93)	0.094	0.16	(−0.47, 0.79)	0.618			
Disclosure gene test results/years (*n* = 98)	−0.11	(−0.25, 0.04)	0.140	−0.11	(−0.25, 0.04)	0.141			
Monitoring coping style per 10 (*n* = 99)	−0.33	(−1.17, 0.51)	0.436	−0.11	(−0.93, 0.72)	0.798			
Situation specific self-efficacy (*n* = 100)	−0.48	(−0.93, −0.04)	0.035	−0.47	(−0.97, 0.03)	0.064			
Time	−0.32	(−0.88, 0.24)	0.264	−0.31	(−0.89, 0.28)	0.296			

*Abbreviations*: *b* estimated regression coefficient, *CI* confidence interval, *FDR* first degree relative, *SDR* second degree relative, *BOC* Breast or ovarian cancer

^a^ Number of subjects having completed the whole dataset on at least one occasion during the data collection time

^b^ Final model: After backward stepwise selection from fully adjusted model at significance level 0.05

^c^ F-test

## Discussion

The mean level of anxiety symptoms among our participants was higher (6.2) compared with earlier studies among women undergoing genetic counseling for hereditary cancer (5.0) [18]. However, the level decreased significantly by post-GPE follow-up, while the mean level of depression symptoms was low and stable throughout the study period. One of the main findings from this study was that lower symptoms of anxiety were associated with longer time since disclosure of the gene test results, higher levels of self-efficacy and having experienced the death of a close relative due to breast or ovarian cancer. While a higher level of education and experienced the death of a close relative due to breast or ovarian cancer was associated with a lower level of depression.

All respondents were satisfied with the GPE course and a majority reported that their knowledge had increased. This might reflect the quality of the course and the group setting. Alternatively, these women were self-selected (after being invited) to attend the course and we must assume that individuals who are uncomfortable with group-based courses would not enroll in such an intervention. This possibility is further enhanced by the fact that our respondents seemed to be quite resourceful in the sense that they were highly educated, a majority cohabitated, they reported high levels of self-efficacy, and they scored high on monitoring coping style.

The mean levels of anxiety and depression symptoms were below the cut-off score (≥8) [39] both before and after attending the GPE course. However, it should be noted that the mean level of anxiety symptoms in our sample was quite high compared with the general population in Norway (4.3) [44], and significantly higher than reported in earlier studies of individuals seeking genetic counseling for hereditary cancer [18]. In the present study, the level of anxiety symptoms was comparable to newly diagnosed breast and ovarian cancer patients (6.8) [45]. Although the mean level of symptoms of anxiety decreased significantly from baseline to posttest, the mean level at posttest was still similar to other cancer groups [46]. Signing up for a GPE course might also increase the perceived anxiety level at the time, because the participants may reactivate thoughts and experiences not fully addressed previously.

The relatively high level of anxiety symptoms observed in our sample might be explained by the fact that all of our participants were mutation carriers. Earlier research has shown that the level of anxiety symptoms after genetic counseling for hereditary cancer is related to a high level of pre-test anxiety and being a mutation carrier [6, 47]*.* Others like Reichelt et al. [48] did not find the same association between being a mutation carrier and increased level of anxiety. However, they found higher HADS-A among women with cancer disease [48]. We did not find support for the latter in our regression model.

The mean time since disclosure of the gene test result was about 5 years, but this varied a great deal (from 1 month to 15 years). The GPE courses we evaluated in the present study were the first to be organized in this health region and many of the eligible participants had lived with the knowledge of their mutation status for a long time. Some might miss someone, like a peer, to talk to about the challenges associated with being a *BRCA* mutation carrier. This again could lead to increased anxiety symptoms. Earlier research has also shown that women with *BRCA* mutations feel alone with their concerns about being a mutation carrier and report that they want to discuss their challenges with peers and health professionals [16, 17]. The latter is indeed what they get in a GPE course and might explain the significant reduction in their anxiety symptom levels.

One should also note that our participants reported a relatively high level of monitoring coping style, which did not surprise us. After all, these respondents were highly self-selected and we would expect a monitoring personality trait to seek and prefer GPE courses such as ours. Previous studies have shown that individuals with high monitoring coping style who seek genetic counseling report higher anxiety before the visit, and that their level of anxiety decreases after the counseling session [27]. We might interpret that a person with a high monitoring coping style experiences a decrease in anxiety level when given the extensive information provided through a GPE course.

The mean depression symptoms score in our sample was low and stable across the observation period. This depression level is similar to a Norwegian population sample and in line with earlier research on genetic counseling [18, 44]. We cannot rule out that one possible explanation of the low depression scores in our study were caused by a selection bias: all the participants enrolled in the course after being invited by the medical genetics department. Common symptoms of depression such as lack of energy and initiative may reduce the probability of signing up for a GPE course, and this will subsequently favor participants with lower levels of depression.

The mixed linear model showed that time since disclosure of test results and higher levels of efficacy were associated with a lower level of anxiety symptoms. It has been well established that time since a potentially traumatic event is associated with improved psychosocial outcome. The women in this study had been enrolled in surveillance programs, some several years previously, and one may presume that they have adjusted to the knowledge of their increased cancer risk. The association between higher levels of self-efficacy and lower anxiety symptoms is consistent with earlier findings [18]. Self-efficacy as a psychological resource is associated with better outcomes in most demanding situations and this may partly explain the association we revealed. More specific medical information will give these individuals higher degrees of satisfaction and further reduce their level of anxiety symptoms [41, 49, 50].

More surprising, we found that having lost a first and/or second degree relative to cancer seems to be a “buffer” for both anxiety and depression symptoms. This is inconsistent with earlier findings [22, 23] and may be explained by the high levels of both self-efficacy and monitoring coping styles. Those who sign up for a course such as ours are more likely to have lived through the grieving process and actively processed it in an adaptive manner. It should be noted that women who experience having their mother diagnosed with breast cancer at a young age, for example, make decisions regarding genetic evaluation and follow-up earlier than women without such an experience [8]. Important life experiences like this may motivate patients to take health preventive actions. As expected, and in consistency with earlier findings, we also found an association between higher education levels and lower levels of depression symptoms [44].

In the present study, the drop-outs were significantly younger and fewer of them had children. We know from earlier studies that younger patients attending genetic counseling for hereditary cancer are often more anxious and vulnerable [51] and this may explain why these participants tend to drop-out. On the other hand, this might be because the youngest felt that the course themes were not relevant to them. A surveillance program, prophylactic surgery, and family issues such as whether their children have inherited their germline mutation may be irrelevant at their current life stage.

## Conclusion

The present study revealed that participants in a GPE course were resourceful in the sense that they had high levels of education, were cohabiting, were highly self-efficacious, and had a high monitoring coping style. Furthermore, the respondents seem to benefit from attending the course which may indicate that the GPE course has the intended effect, and therefore could be a valuable supplement to traditional genetic counselling. Finally, we identified some participants who may be more vulnerable and therefore should receive greater attention; specifically, those who were newly diagnosed with a *BRCA* mutation, who had lower levels of self-efficacy, or who had lower levels of education. A consequence of these findings should include arranging regular GPE courses and offering them as part of the genetic follow-up. In our department, all women carrying a *BRCA* mutation are now offered a GPE course within 6–12 months after disclosure of the gene test results.

## Abbreviations

BGCSES: Bergen Genetic Counseling Self-efficacy Scale
GPE: Group-based patient education
HADS: Hospital Anxiety and Depression Scale
MRI: Magnetic resonance imaging
SD: Standard deviation
TMSI: Threatening Medical Situation Inventory
